# Integration of Proteomics, Bioinformatics, and Systems Biology in Traumatic Brain Injury Biomarker Discovery

**DOI:** 10.3389/fneur.2013.00061

**Published:** 2013-05-31

**Authors:** J.D. Guingab-Cagmat, E.B. Cagmat, R.L. Hayes, J. Anagli

**Affiliations:** ^1^Banyan Biomarkers, Inc., Alachua, FL, USA

**Keywords:** proteomics, biomarkers, traumatic brain injury, bioinformatics, systems biology

## Abstract

Traumatic brain injury (TBI) is a major medical crisis without any FDA-approved pharmacological therapies that have been demonstrated to improve functional outcomes. It has been argued that discovery of disease-relevant biomarkers might help to guide successful clinical trials for TBI. Major advances in mass spectrometry (MS) have revolutionized the field of proteomic biomarker discovery and facilitated the identification of several candidate markers that are being further evaluated for their efficacy as TBI biomarkers. However, several hurdles have to be overcome even during the discovery phase which is only the first step in the long process of biomarker development. The high-throughput nature of MS-based proteomic experiments generates a massive amount of mass spectral data presenting great challenges in downstream interpretation. Currently, different bioinformatics platforms are available for functional analysis and data mining of MS-generated proteomic data. These tools provide a way to convert data sets to biologically interpretable results and functional outcomes. A strategy that has promise in advancing biomarker development involves the triad of proteomics, bioinformatics, and systems biology. In this review, a brief overview of how bioinformatics and systems biology tools analyze, transform, and interpret complex MS datasets into biologically relevant results is discussed. In addition, challenges and limitations of proteomics, bioinformatics, and systems biology in TBI biomarker discovery are presented. A brief survey of researches that utilized these three overlapping disciplines in TBI biomarker discovery is also presented. Finally, examples of TBI biomarkers and their applications are discussed.

## Introduction

Tremendous efforts have been put into the discovery of biomarkers that can diagnose disease or injury. A quick search for scholarly articles that include the word biomarker can yield more than half a million hits. However, the overall status of biomarker development and clinical validation is very disappointing. There are only a handful of novel biomarkers that are of clinical relevance, and the rate at which a biomarker is introduced to the market is dismal. One estimate shows that since 1998, new protein biomarkers that were approved by the US Food and Drug Administration fell to one per year (Rifai et al., 2006). The reasons for this trend are numerous and one strategy to reverse the fall is a better understanding of the whole process itself. One key strategy in hunting for that robust biomarker is the combination of scientific disciplines. In traumatic brain injury (TBI), an interdisciplinary approach is employed by combining the methods and tools from three fields, namely, proteomics, bioinformatics, and systems biology.

Proteomics is the study of protein populations or proteomes. The term “proteome” was coined in 1995 (Wasinger et al., 1995) to describe the protein complement of a genome. This came after realizing that at least half of the proteins encoded by the human genome have no known functions. The move to study the message (mRNA or cDNA) and focus on the product of the message (proteins) gave birth to proteomics. Proteomics assesses the expression level of proteins, post translational modifications and interactions of proteins within a tissue, cell, subcellular compartment, or biofluid. The goal is to obtain a large scale and a global view of physiological conditions and disease processes. However, studying the global systems of proteins has produced a large amount of data, and making sense of the complex data generated became a problem. In the beginning, it was clear that processing a vast amount of data requires the aid of computers. Like genomics a decade ago, proteomics tackled the problem by enlisting the help of bioinformatics and later on, systems biology.

Bioinformatics combines mathematics and computer technology to deal with the analyses of large numbers of proteins while systems biology unveils the global network of physiological environments. Bioinformatics has become an integral part of proteomics, strategically mining data for sensible results. Systems biology on the other hand tries to look at the big picture by mapping interactions of isolated proteins, akin to looking at the ecosystem of the whole forest, rather than just the individual trees.

The triad of proteomics, bioinformatics, and systems biology has been applied to study protein behaviors in myriad disease pathologies. It was no different with neurological conditions such as TBI, Alzheimer’s disease, and stroke. Neuroproteomics (Choudhary and Grant, 2004), a field under the proteomics umbrella, has zeroed in these disorders, extracting insights into the dynamics and interactions of proteins in these disease states (Ottens et al., 2006, 2010; Bayes and Grant, 2009; Alzate, 2010; Shoemaker et al., 2012).

One of the neurological conditions that received a fair amount of media attention lately is TBI. Although TBI is known as the “silent epidemic,” the public is beginning to be aware of the injury as war veterans come home from war-zone blasts. Even the military has acknowledged that TBI is the “signature injury of the conflicts in Iraq and Afghanistan” (Risdall and Menon, 2011).

Increasing media coverage to concussive injury has increased lately. This is partly due to the increase in suicides of football players. High profile cases of professional football players have captivated the public, highlighted by the suicide of Dave Duerson. Mr. Duerson, a professional football player, was suspected to have suffered TBI during his playing years. He was found dead with a gunshot to his chest, not in his head, to preserve his brain for science.

Statistically, TBI is one of the leading causes of disability in the United States. It is considered one of the major health problems annually claiming 5% of the lives of the two million victims. Around 25% are hospitalized and approximately half are treated and released after emergency care (Smith et al., 2003; Johnson et al., 2004). It is estimated that by 2030, the public health impact of TBI will increase (Mathers and Loncar, 2006). This should alarm us as road traffic accidents will be the most common cause of blunt trauma, making TBI the fourth leading cause of disability.

The disturbing reality for victims and their families is that currently, there are no FDA-approved treatments or therapy (except for pain relievers) that can alleviate the effects of TBI. One of the most pressing needs however, is the accurate diagnosis and monitoring of patients. Physicians should be guided if patients respond to the treatment and improve. But to this day, clinicians are limited only by parameters such as brain pH, pO_2_, intracranial pressure (ICP), and temperature. Brain imaging techniques such as Computer Tomography (CAT) and Magnetic Resonance Imaging (MRI) scans have provided information of damaged regions non-invasively, but only looking at the injury in a short time. The limitations of traditional diagnosis have hindered the overall progress in understanding the condition, highlighting the need for more accurate diagnostic tools. The goal is that a robust biomarker or panels of biomarkers will complement existing diagnosis, and eventually replace the more traditional ones.

In this paper, advances and limitations of proteomics, bioinformatics, and systems biology will be discussed. We shall then try to integrate the three fields in relation to biomarker discovery, and limiting the discussion only to protein biomarkers in TBI. This article is structured as follows. In Section “Biomarkers, TBI Models, Proteomics, Bioinformatics, and Systems Biology, Their Definition,” we define biomarkers, TBI animal models, proteomics, bioinformatics, and then systems biology. In Section “Protein Profiling,” we shall review the methods, challenges, and technical difficulties inherent in identifying proteins. Section “Biomarker Applications” deals with the present panels of proteins that can be used as a biomarker for TBI.

## Biomarkers, TBI Models, Proteomics, Bioinformatics, and Systems Biology, Their Definition

Biomarkers are indicators of normal biological processes or disease states. A biomarker can also be a gage of pharmacological response in therapeutic interventions (Lesko and Atkinson, 2001).

The idea in biomarker discovery is that organs secrete specific molecules that can indicate a physiological malfunction. In general, these are any biomolecules that can serve as a fingerprint showing up from samples of affected tissue or peripheral fluids of the affected area. In the context of TBI and proteomics, ideal biomarkers are proteins that are only present in the brain, leaked out from the blood brain barrier and into the person’s blood or cerebrospinal fluid (CSF) during or after brain injury. These molecular signatures should be proportional to the impact and the extent of damage in the brain, and should reflect differences between age groups and sex.

Numerous animal models of TBI have been developed to understand the heterogeneous nature of brain injury (recently reviewed by Chopp et al.) (Xiong et al., 2013). Due to their low cost and the presence of more standardized outcome measurements, rodent models are particularly used to study TBI although bigger animals are closer to human physiology. Controlled cortical impact (CCI) uses a controlled degree of impact by a pneumatic or electromagnetic impact device (Lighthall, 1988; Dixon et al., 1991). Penetrating ballistic-like brain injury (PBBI) model mimics severe to moderate TBI such as gunshot wounds. PBBI is induced by transmission of high energy projectiles and a leading shockwave producing a temporary cavity in the brain that is many times the size of the projectile itself (Williams et al., 2005). Another widely used model is the fluid percussion injury (FPI) where a contusion force is incurred by the movement of a fluid in a chamber. In the drop-weight impact acceleration injury, the skull (with or without craniotomy) is exposed to a weight that is dropped from a certain height and injury severity can be altered by adjusting the mass of the weight and the height from which it falls. The more recent TBI models are the blast models that mimic TBI induced by explosive devices. Blast-induced brain injuries have been predominant among military personnel who have been exposed to a blast but do not have external injuries (Warden, 2006; Benzinger et al., 2009). Different variations of blast TBI animal models have been developed to elucidate the effects of primary blast waves on the brain (Wang et al., 2005; Cheng et al., 2010; Svetlov et al., 2010; Risling et al., 2011). Elucidation of the mechanisms of blast injury, identification of biomarkers and, eventually, the development of strategies for mitigating blast-induced brain injury will benefit from further design optimization, characterization, and standardization experimental parameters of blast TBI models.

While TBI can occur as a result of auto accidents, violence, or sports injuries it has left the shadows with the war in Iraq and Afghanistan. Twenty-first century warfare exposes military personnel to blast injuries resulting from high order explosives. The Kevlar helmet, although an excellent protection against penetrating brain injury, offers little protection from blast injuries (Lew, 2005; Okie, 2005). Accurate statistics are not currently available, but it is estimated that more than 50% of all casualties from the Afghanistan and Iraq theaters have sustained head injuries (Warden, 2006) compared to 15–25% from twentieth century conflicts (Carey, 1996). Of the 1.4 million TBIs that occur annually, the vast majority, between 75 and 90% are mild or moderate (mTBI) (Jager et al., 2000; Gerberding, 2003). Mild and moderate TBI, also called concussion, occurs when an impact or forceful motion of the head results in a brief alteration of mental status, such as confusion, disorientation, brief memory loss, or brief loss of consciousness. Because they produce a number of imprecise perceptual symptoms without diagnosable objective structural brain alterations, mTBIs are challenging to diagnose (Lyeth et al., 1990; Hamm et al., 1993; Kibby and Long, 1996; Margulies, 2000). Furthermore, many sufferers fail to recognize the potential severity and seriousness of their injury thus do not seek medical attention (Alexander, 1995; Kushner, 1998). TBI is thus under-diagnosed and under-represented in medical statistics. However, even brief alterations in mental status can inflict profound and persistent impairment of physical, cognitive, and psychosocial functioning (Binder, 1997; Ruff and Jurica, 1999). Furthermore, TBI is an epigenetic risk factor for Alzheimer’s and Parkinson’s diseases (Smith et al., 2003; Szczygielski et al., 2005). Although TBI is a major focus of casualty care in combat areas and the principal cause of mortality and morbidity due to improvised explosive devices (IEDs) and other hazards, there are no FDA-approved pharmacologic therapies that have been demonstrated to improve functional outcomes.

Proteomics is defined by many as the study of the protein complement of the genome, the proteome (Blackstock and Weir, 1999; Stults and Arnott, 2005). The proteome is the set proteins from the whole organism or specific organ at specific physiological conditions.

Several definitions of bioinformatics can be found in the literature today. What suits us is the idea that bioinformatics is a tool to mine vast amounts of data using computer technology and mathematics (Hagen, 2000; Kumar and Mann, 2009).

Systems biology came into picture as soon as proteins were identified from proteomics experiments. For example, low concentration proteins can now be identified in an injured brain; however, a list of individual proteins may not make sense. To understand the connections of isolated proteins, systems biology came in.

The science of systems biology is still considered to be in its infancy and a consensus on its definition has not been fully reached (Ideker et al., 2001; Kitano, 2002a,b; Chuang et al., 2010). For us, it is an approach to study the complex interactions of biological systems. It examines, assembles, and maps the properties and regulations of tightly interconnected biological systems.

## Protein Profiling

Identification of proteins is one of the main goals of biomarker discovery. The conventional method of identifying proteins as a marker for disease is by measuring a specific compound known to be part of the pathophysiology. In TBI for example, the presence of glial fibrillary acidic protein (GFAP) in the blood can mean damage to the glia. Also, tau and spectrin protein breakdown products in the blood indicate damage to the axons. One can also examine the unregulated breakdown products of necrotic cell death. Breakdown products of calpain mediated proteolysis can be used as biomarkers of TBI. This is the same for products of apoptotic cell death, from the activation of caspase (Büki et al., 1999; Farkas et al., 2005; Svetlov et al., 2009; Risdall and Menon, 2011).

A novel method, which is the subject of this review, is the data driven and high-throughput approach of discovery. In this strategy, the samples from normal and TBI patients are compared, screening for differences between the two. This approach consistently uses mass spectrometry (MS) and most of the time it is *discovery driven* instead of being *hypothesis driven* (Stults and Arnott, 2005). In discovery driven types of experiments, information is collected and then patterns are sought. Unlike hypothesis driven research that disproves or proves a defined hypothesis, discovery driven research collects a huge amount of information first then extracts questions and answers from lots of data. It may sound like a “blind shot” to find answers, but our current technology enables us to do this. If history is a good indicator, it worked with genomics and metabolomics, so performing discovery driven experiments with an entire proteome is logical.

### Historical background

It was almost 40 years ago when two-dimensional electrophoresis was invented and described in a paper (O’Farrell, 1975), giving way to the separation of more complex mixtures. A few years after, in the early 1980s, the first profiling of human CSF (Merril et al., 1983) and mammalian brain (Klose and Feller, 1981) were reported. These started the systematic classification of proteins from the brain. By the mid-80s, the first proteomic database SWISSPROT was established (Bairoch and Boeckmann, 1991; Bairoch and Apweiler, 1997; Peitsch et al., 1997). In the end of that decade, two ionization techniques for MS analysis were introduced, making large protein analyses possible (Karas and Hillenkamp, 1988; Fenn et al., 1989). High-throughput and gel free proteomics came into being when liquid chromatography (LC) was integrated with MS around 1996 (Appella et al., 1995).

Ten years later, the profile of a mouse’s brain was created, identifying 7,792 proteins (Wang et al., 2006), ranging in abundance from tens of copies to hundreds of thousands of copies.

### MS-based neuroproteomics workflow

In a typical neuroproteomics experiment, proteins from the brain or spinal cord tissue are extracted as a mixture of proteins. Depending on the experiment, obtaining the proteins can be done with tissue homogenization, cellular fractionation, or affinity fractionation. Then the complex mixture is further separated to reduce its complexity.

Three of the common separation tools for protein separations are two-dimensional electrophoresis (2DE), one-dimensional electrophoresis (1DE), and a two-dimensional LC. In the more common bottom-up proteomics, after subjecting a sample to one of the three methods mentioned above, the proteins are then digested by an enzyme prior to analysis by MS. After protein separation and digestion, the resulting peptide mixture is further resolved by a nanoflow liquid chromatography (nanoLC) based on the peptides hydrophobicity prior to introduction into the mass spectrometer by nano-electrospray ionization. Many TBI proteomic biomarker studies have relied on the bottom-up approach. Putative protein biomarker candidates were identified in rat CCI model using 1D-SDS-PAGE prior to bottom-up proteomic analysis (Will Haskins). An improved two-dimensional platform employing a protein pre-fractionation step by cation-anion exchange and ID-SDS-PAGE prior to bottom-up proteomic analysis was used in subsequent TBI biomarker studies from our group (Kobeissy et al., 2006; Ottens et al., 2007). Kochanek’s group was the first to use 2D-PAGE in TBI biomarker study (Jenkins et al., 2012). Siman et al. (2004) performed MALDI-MS following 2D-PAGE of proteins released from TBI cell culture model to identify acute TBI protein biomarkers. 2D-PAGE and mass spectrometric analysis have been implemented in oxidative stress TBI biomarker studies (Opii et al., 2007). An alternative to the above approach is shotgun proteomics (Wolters et al., 2001; McDonald and Yates, 2002, 2003; Wu and MacCoss, 2002). The complex mixture in shotgun analyses is directly digested without prior separation or fractionation. Variations of this method exist but all shotgun proteomics begins with a mixture of proteins. For example, a complete protein digest without prior separation can be separated by LC and then analyzed by MS in real time. A shotgun proteomic approach based on nanoLC in conjunction with matrix-assisted laser desorption/ionization time of flight tandem MS (MALDI-TOF MS/MS) was utilized to quantitatively analyze the protein content of consecutive ventricular CSF samples of severe TBI patients (Hanrieder et al., 2009). Recently, our group has applied shotgun proteomics to profile the neuronal-glial biomarkers released into conditioned media collected from MTX-, NMDA-, and STS-treated cell cultures (Guingab-Cagmat et al., 2012).

One application of MS is in the identification of intact proteins (i.e., without enzyme digestion) referred to as top-down approach. In the context of proteomics, top-down is an emerging technology but more difficult to implement compared to the more widely used bottom-up approach. For proteomics, top-down has the advantage of preserving the forms of proteins present *in vivo* by measuring them intact, rather than measuring peptides produced from them by proteolysis. This approach is particularly useful in characterization of post translational modifications which may be challenging to analyze with enzymatic digestion. But in order to perform this kind of analysis, an expensive instrumentation is a requirement. Most of the laboratories however don’t have the luxury of having a Fourier Transform Ion Cyclotron Resonance Mass Spectrometer (FT-ICR-MS) (Marshall et al., 1998; Shi et al., 1998), or the relatively less expensive Orbitrap mass spectrometer (Thermo’s Orbitrap Elite) or access to these kinds of instruments. The very advantage of these kinds of instruments is that they are highly sensitive and capable of ultra-high resolution. The downside however is that maintenance of FT-ICR-MS is very expensive since it requires cooling a very strong magnet, on top of an expensive machine. The Orbitrap mass analyzer traps ions using an electrostatic field, instead of a magnet. The cost and maintenance are now relatively lower, but still an expensive machine. Possibly due to these reasons, top-down proteomics is yet to be accepted and widely implemented to TBI studies.

Presently, technologies that focus on identifying less abundant proteins are gaining traction. These methods are usually based on MS, and the requirement is that a step prior to injection into the mass spectrometer is added. Broadly, the steps prior to MS can be categorized into chemical modifications and direct enrichments. An example of chemical modification is affinity tagging. A popular tagging method is ICAT or isotope coded affinity tags. It is used to quantify and identify plasma biomarkers of TBI. These kinds of experiments can identify several candidate proteins, from tens to hundreds. Protein biomarkers in serum of pediatric patients with severe TBI were identified by ICAT-LC-MS/MS (Haqqani et al., 2007a,b). Another approach is isobaric tagging for relative and absolute quantification (ITRAQ). An example is the study by Crawford et al. (2012) on the identification of protein markers of TBI outcome. Here, CCI mouse model was used to identify plasma biomarkers specific to mild or severe TBI at 24 h, 1 month, or 3 months post-injury. In addition, they used apolipoprotein E 3 and 4 transgenic mice, which demonstrate relatively favorable and unfavorable outcomes respectively, following TBI to identify proteins that are significantly modulated in response to the TBI*APOE genotype interaction representing potential prognostic biomarkers. ITRAQ has also been applied in the identification of serum biomarkers and demonstrating their potential for predicting elevated intercranial pressure in TBI patients (Hergenroeder et al., 2008).

Direct enrichment entails some separation prior to MS analysis. These separation strategies usually apply chromatography, SDS-PAGE, or antibody. For example, cation-exchange chromatography, SDS-PAGE, and then LC, were performed on a rat CCI model to identify putative protein biomarkers post 48 h TBI (Kobeissy et al., 2006; Ottens et al., 2007). The results included 59 differential protein components of which 21 decreased and 38 increased in abundance after TBI. Proteins with decreased abundance included collapsing response mediator protein 2 (CRMP-2), glyceraldehyde-3-phosphate dehydrogenase, microtubule-associated proteins MAP2A/2B, and hexokinase. Conversely C-reactive protein, transferrin, and breakdown products of CRMP-2, synaptotagmin, and alphaII-spectrin were found to be elevated after TBI.

### Bioinformatics in identifying proteins

Proteomics experiments to identify proteins are tedious. It is akin to breaking a huge and complicated puzzle and then putting the pieces together again. With our puzzle analogy, manual integration of the pieces (smaller peptides) is impossible to complete without the help of computers.

The need for algorithms to identify proteins married bioinformatics to proteomics. Once the developers of algorithms were on board, they needed to know some of the rules of protein science. One of these is for example in sample digestion. If trypsin was used in the digestion, this enzyme is known to *only* cleave proteins after both lysine and arginine, *as long* as the next amino acid sequence is not proline (Fraser and Powell, 1950). This and many other rules have to be grasped by software developers.

Other rules that computer scientists should fundamentally understand are mass spectra. Historically speaking, MS of digested proteins was performed predominantly by matrix-assisted laser desorption ionization time of flight or MALDI-TOF. MALDI (Karas and Hillenkamp, 1988) is a kind of ionization that is regarded as “soft,” enabling large biomolecules to be ionized and carried to the mass analyzer. The ionization requires two things: the energy from the laser and the matrix. Although the mechanism of MALDI is still in question, it is believed that the ionization of the analyte happens after the matrix absorbs the energy from the laser, the matrix imparting the energy to the analyte, thereby ionizing the sample.

Once the calculated experimental spectrum or mass lists are produced, these are matched against a protein database. Another set of spectrum, a theoretical one, is also matched to the database. Theoretical and experimental results are compared and computed, to have confidence in the identified proteins (Maggio and Ramnarayan, 2001; Colinge and Bennett, 2007; Matthiesen, 2007; Webb-Robertson and Cannon, 2007).

The above method, performed in MALDI-TOF, is commonly referred to as peptide mass fingerprinting (PMF). PMF’s requirement is that a single spectrum should contain the peptides of the protein. The introduction of LC and ESI however removed the single spectrum requirement. With LC experiments, identification of peptides became more challenging.

In a typical LC-MS/MS analysis, one can predefine the number of the most intense peaks to be selected for dissociation. For example, in our laboratory, we subject the 10 most intense peptide signals to tandem MS (MS/MS) fragmentation (data dependent scanning). Every second, the MS analyzes the sample and produces a full MS Scan of *∼*20,000 intact peptides. Based on the initial full MS scan, the mass spectrometer, following the user’s settings, selects again and fragments up to 10 distinct peptides, producing another set of MS/MS spectra.

During the selection of peptide however, the same peptide can be selected more than once. To avoid this problem, a dynamic exclusion strategy is usually implemented. For example, if a peptide was selected three times already over the span of 18 s, that peptide is placed in the exclusion list for the next 25 s. The cycle of subjecting the 10 most intense peptides to MS/MS and the production of s full MS scan is repeated until the chromatography is done.

Tandem MS (MS/MS) provides an additional degree of information in identifying proteins. One can see that in a single analysis, a large number of MS/MS spectra are produced. Assigning the peptide sequences responsible for the generation of the observed fragments is challenging. Since the fragmentation process in MS/MS follows some rules, rules that software developers exploit, it is now possible to identify proteins that are subjected to tandem MS.

Collision of an inert gas with large proteins (such as collision induced dissociation or CID) fragments the proteins apart into smaller peptides. This happens inside the trapping cell of the MS. The breaking of proteins follows a certain type of fragmentation pattern (most researchers follow the nomenclature introduced by Roepstorff and Fohlman, 1984). It is widely known that proteins in the gas phase can break into set of ions (“*b*, *y*, and *a*” type ions) (Bencsath and Field, 1988; Polfer et al., 2005; Liu and Schey, 2008; Chen et al., 2009; Paizs and Mann, 2012).

Even though the rules of producing specific ions are clear, problems still exist. Some compounding factors happen when there can be some additional peaks resulting from neutral losses (*b*-H_2_O, *y*-H_2_O), ammonia loss (*b*-NH_3_), from contaminating peptides, small molecules, or even missing peaks. Some peaks can be shifted due to amino acid modifications. And as in any other analytical signal, the presence of noise even complicates the spectrum interpretation. These hinder the peptide sequence assignment to each spectrum.

### Software for peptide and protein identification

The process of protein identification benefited from the maturation of two technologies, the computer hardware and database software. Protein database search has become a powerful approach to address the challenge of protein ID. Currently, numerous bioinformatics software for computational peptide identification from MS/MS data are available in the market (Xu and Ma, 2006).

The first computer program to use a database search was Sequest. Acquired by Thermo Scientific and commercially available through Proteome Discoverer (Thermo, San Jose, CA, USA. www.thermo.com), the development of this software can be traced back to Yates et al. (1995) in the early 1990s at the University of Washington. The scoring function in this package is heuristic in nature, and it was considered to be the first really useful bioinformatics technique in the field of proteomics. The software integrates correlational analysis between data dependent mass spectral scans and a FASTA protein database. Sequest searches and identifies peptides and the corresponding modifications that the user specifically queries. Using these peptide identifications, one can make inferences about the proteins in the sample.

In Sequest, the first process is the extraction of tandem mass spectra from the raw file. Theoretical candidate sequences from the digested proteins in the database are listed. Within a defined tolerance set by the end user, the algorithm determines which one matches the experimental peptides’ molecular weight. A comparison of the candidate’s *b* and *y* ions to the experimental spectra are made and scored as primary score (Sp). The primary score sorts the candidate sequences in descending order. Sequest uses two scoring functions, so after the initial candidate sequence is determined; the top peptides are taken off the list. A second function rescores the hits by computing a cross correlation, taking into account their height and mass position. The new candidates are resorted in descending order. After taking into account the possible random matches, the final list after resorting is the final SEQUEST scores (Xcorr). These top hits are reported back and stored into the search files (.msf). In addition to Sequest’s Xcorr, users can export several other parameters such as Sp or DeltaCn. DeltaCn measures how good the XCorr is relative to the next best match. Overall, Xcorr is a robust measure of how accurate the match was between theoretical and experimental peaks.

Several algorithms came after Sequest. MASCOT (Perkins et al., 1999) and X! Tandem also became popular search engines. Owned by Matrix science, MASCOT is commercially available, although the scoring has never been patented or published. With MASCOT, the accuracy score is probability based. This is measured by Ion score, and a *P*-value gives a relative score. On the other hand, X! Tandem is an open source tool. These search engines approach the problems differently and uses different algorithms. With X! Tandem, hyper score and *E*-value are two of the parameters calculated.

In some instances however, there are situations that a protein database is not yet available. This can happen in the analysis of an organism where its genome sequence is incomplete or unavailable. In addition, if one is only interested in identifying novel isoforms of the protein, often, the database is unavailable. A popular approach to tackle the problem is to perform a *de novo* (Shevchenko et al., 2001) sequencing. Spectrum identification in *de novo* analysis uses a database of candidate peptides consisting of all possible linear amino acid sequences (Xu and Ma, 2006). This method can be used also for searching peptide homologs and modifications. In the early days of the development of algorithms in *de novo* sequencing, researchers in this field attempted to reconstruct peptide sequences by making all of the amino acid combinations. This was not applicable though due to generic problems. However, the market has seen software for *de novo* sequencing. Algorithms in *de novo* sequencing usually filter the experimental mass list to remove noisy peaks. PEAKS (Ma et al., 2003) and PepNovo (Frank and Pevzner, 2005) are some of the software that facilitate fast *de novo* peptide identification. A hybrid between the *de novo* sequencing and protein database searching is known as tag-based approach. Sequence-tagging uses the *de novo* analysis to identify subpeptides or sequence tags hypothesized to occur in the sequence. In these kind of experiments, information is usually extracted from database that contains the tags (Mann and Wilm, 1994).

Since the sequencing results of *de novo* shows a close resemblance compared to the output of known protein database, *de novo* is usually used in validating the accuracy of database-derived protein identifications (Shadforth et al., 2005). Validation of the accuracy of one’s result is one of the issues that are tackled by end users and software developers. Reviewers of top proteomic journals have pushed to address this issue. This will be discussed next.

### False detection rate

False detection rate (FDR) measures the false positive proteins identified. FDR provides a statistically meaningful estimate of the uncertainty in protein identification. It is usually a good validation, for example in large data sets of brain proteins. Most proteomic journals require FDR to be reported. In measuring FDR, a decoy database is usually used. Decoy database for FDR calculations were pioneered by Gygi and co-workers, in which decoys consist of a randomized or scrambled sequence database (Elias and Gygi, 2010). The parameters used in regular search are applied to the decoy database search. Matches using decoy database search is not expected to be significant, and the number of matches found in a decoy search is a good estimate of the real FDR in the regular forward sequence database search.

Although there are two ways to implement a search in a decoy database, users preferentially use one from the other. The most preferred method is the concatenated approach. In this method, the decoy and the non-decoy databases are linked together.

The other method is a more conservative approach. The search of MS/MS data is separate from non-decoy to decoy databases and the number of matches for each database is counted.

### Systems biology

After a database search and identification of proteins, usually a huge library of information is generated. The next step is to know the protein’s functions and the connections of these identified proteins. Rather than focusing on individual molecular components, systems biology seeks to understand the dynamics that govern protein networks, the functional set of proteins that regulate cellular decisions related to TBI. From the perspectives of drug discovery and diagnostics, systems biology gives important and practical clues concerning the pathways relevant to brain injury and the effects that drugs might have on them. Therefore, it enhances the entire biomarker and therapeutic drug discovery, development, and commercialization process (cite Systems bio approach/Theranostics). Recently, protein biomarkers of TBI, induced by penetrating ballistic-like injury model (PBBI), were identified by the proteomics followed by systems biology analysis (Boutté et al., 2012). These proteins are ubiquitin carboxyl-terminal isozyme 1, tyrosine hydroxylase, and syntaxin-6. Using semi-quantitative western blotting analysis, the said proteins were found to be elevated after 72-h post-injury compared to control. It should not be a surprise that Ubiquitin carboxy-terminal hydrolase L1 protein (UCHL1) is already in clinical trial as a biomarker.

The connections or network of connections are pictured using nodes and links. The nodes can be a biomolecule, such as proteins or DNA. The link or the connections between these nodes represent the biochemical interactions or the connections can highlight relationships between nodes, such as the strength of predicted binding or physical interactions. Theories in the science of systems study and statistical mechanics, in conjunction with graph theory, can be applied to glean insights about the network. Mapping the connection of these proteins is the driving force of pathway-based biomarker discovery and diagnosis. Particularly in TBI, upregulated proteins after the injury are hunted and identified as possible diagnostic biomarkers. Numerous scientific publications containing networks of cellular pathways are scattered throughout archives and available data are growing fast. Historically, most of the repositories of large scale sequencing projects were mostly nucleic acid and amino acids. But this gave way to other biomolecules such as proteins. Lately, databases that store proteins have been steadily increasing. For example, the Database of Interacting Proteins can be queried for known protein-protein interactions or PPI (Xenarios et al., 2001).

The nuts and bolts of these bioinformatics software, which systems biology has integrated, are geared toward people with a strong background in computer science and statistics. Since we are the end users of this technology, we will focus on software that we are familiar with and have been using. Readers are directed to other sources of in-depth reviews with regards to systems biology. Three commercially available pathway analysis software include Pathway Studio (Ariadne Genomics, Rockville, MD, USA), Metacore (Thompson Reuters, New York City, NY, USA), and Ingenuity (Ingenuity Systems, Redwood City, CA, USA). These tools enable the identification of the relationship among proteins, small molecules, cell processes, and diseases. Pathway analysis provides information on what is known to interact with the proteins that are identified in the sample as well as association of these proteins to cellular processes.

## Biomarker Applications

Clinically validated biomarkers are needed for the accurate diagnosis of mild TBI. This type of TBI is particularly hard to accurately measure and the situation is, made more challenging by patients who sometimes hide their symptoms. There is no gold standard yet for diagnosing mild TBI (Shenton et al., 2012), not even by conventional assessment through neuroimaging techniques (Niogi and Mukherjee, 2010). The lack of a consensus definition of mild TBI further complicates the matter (Ruff and Jurica, 1999; Arciniegas and Silver, 2001) and the challenge lies in accurate diagnosis in managing post-injury. The Veteran’s Administration Clinical Practice Guideline released a working document on criteria to diagnose mild TBI. These diagnostic criteria include an initial Glascow Coma Scale (GCS) of 13–15; less than a 30-min loss of consciousness; post traumatic amnesia up to 24 h after the injury and alteration of consciousness (Management of Concussion/mTBI Working Group, 2009). Other factors may compound this guideline. In addition to patients trying to hide the true injury, proper diagnosis is compounded by alcohol ingestion, polytrauma, sedatives, pain killers, and drugs of abuse.

A biomarker that is measurable in the blood would be useful in these kinds of situations, where a polytrauma exists. It was suggested that instead of using one biomarker, a panel of biomarkers could be helpful. Mondello et al. (2012a) have explored the ratio of GFAP and UCHL1 concentrations to assess patients with severe TBI.

Another type of injury that needs to be addressed by biomarkers from the blood is in diffuse axonal injury (Inglese et al., 2005). The microstructural axonal damage in this kind of injury is believed to be a challenge to detect by neuroimaging techniques such as computed tomography and conventional MRI.

Drug discovery is one of the fields that will greatly benefit from a signature marker for TBI. New therapeutic development traditionally has an extremely high triage rate because more than 90% of drugs that advance to Phase I clinical trials fail. Some argue such extreme loss can be overcome by guiding all new therapeutic development and clinical trials with a disease-relevant diagnostic test. Discovery of translational biomarker (from animal studies to clinical trials) might help to finally deliver the long sought after clinical trial success. “*Theranostics* represents the convergence between *Thera*peutics and diag*nostics* (Bissonnette and Bergeron, 2006; Hooper, 2006).” It has been viewed as the parallel use of new therapy and diagnostic tests for a human disease or disorder so as to facilitate drug development and clinical trials and to achieve optimal clinical outcomes in a population of patients. Importantly, in recognizing the emerging role of the theranostic approach, the FDA has recently drafted a Drug-Diagnostic Co-Development Concept Paper (Hinman et al., 2006) with the goal of setting guidelines for prospective co-development of a drug or biological therapy (drugs) and a device test in a scientifically robust and efficient way.

One example of a theranostic approach to drug development is a novel biomarker-guided approach in our laboratory that combines calpain-generated acute brain injury-tracking biomarkers with potent and selective calpain inhibitor drug candidates to fast-track and improve the chances of successful drug development for CNS injury. During brain injury, neural proteins or their breakdown products generated by calpains (μ-calpain and m-calpain) are released into the extracellular environment and eventually reach the CSF in relatively high concentration (Wang et al., 2005). In due time the proteins reach the blood stream either via the compromised blood brain barrier (BBB or via filtration of the CSF). Clearance and half-life of the biomarkers contribute to the final concentration that can be measured in the blood. The CSF volume of an adult human (CSF 125–150 mL) is about 30- to 40-fold less than the blood volume (4.5–5 L) which explains why the brain biomarker concentration is significantly higher in the CSF samples versus blood samples and makes the former valuable for drug development. Enabled by recent technological advances in proteomics, novel brain injury biomarkers that have elevated levels in biofluid such as CSF or blood after TBI have been discovered.

## Possible Biomarkers for Traumatic Brain Injury

We now know that despite the efforts in brain injury research to discover and develop disease tracking markers, currently there are no clinically validated biomarkers to diagnose TBI. Even though the search continues, several candidate biomarkers of TBI biomarkers are in the clinical validation pipeline. Extensive studies are being pursued to move these protein biomarkers to clinical validation. The aforementioned techniques in proteomic have been employed in the discovery for candidate biomarkers of TBI. Kobeissy et al. identified 59 differentially proteins 48 h post TBI using a CCI rat model. Proteins that were decreased in abundance included CRMP-2, glyceraldehyde-3-phosphate dehydrogenase, microtubule-associated proteins MAP2A/2B, and hexokinase (Kobeissy et al., 2006). Upregulated proteins included C-reactive proteins, transferrin, and breakdown products of CRMP-2, synaptotagmin, and alphaII-spectrin. Western blotting analysis confirmed the differential changes in the mentioned proteins. This study provided insight into the mechanism of TBI and generated candidate biomarkers that can aid in the evaluation of the severity and progression of injury as well as in the development of possible therapies. The need for strengthening the role of systems biology and its application to the field of neuroproteomics due to its integral role in establishing a comprehensive understanding of specific brain disorder and brain function in general was highlighted in a review by Kobeissy et al. (2008). The use of a systems biology-based approach to drug discovery and development for TBI based on the advances in genomics, proteomics, bioinformatic tools, and systems biology software has been shown (Zhang et al., 2010). Recently, Boutté et al. (2012) conducted a proteomic analysis and brain-specific systems biology in a rodent model of PBBI. In their study, a combination of two-dimensional gel electrophoresis and MS was used to screen for biomarkers in a rat model of PBBI. Brain-specific systems biology analysis of brain tissue identified 321 upregulated and 65 downregulated proteins 24 h post PBBI compared to sham controls. In their gene ontology analysis, the majority of upregulated proteins were cytoskeletal (10.5%), nucleic acid binding (9.3%), or kinases (8.9%). Most proteins were involved in protein metabolism (22.7%), signal transduction (20.4%), and development (9.6%). Pathway analysis indicated that these proteins were involved in neurite outgrowth and cell differentiation. Further confirmation of these proteins was conducted using semi-quantitative Western blotting. Among these proteins that indicated consistent increase in the brain tissue and CSF at several time points post PPBI were UCHL1, tyrosine hydroxylase, and syntaxin-6. Antibody-based platforms, antibody microarrays (AbMA), and reverse capture protein microarrays (RCPM) complementing the classical methods based on 2D gel electrophoresis and mass spectrometry (2DGE/MS) has been proposed for discovery of potential biomarkers for blast neurotrauma (Agoston et al., 2009). Kwon et al. (2011) combined behavioral, proteomics, and histological studies to investigate stress and blast-induced TBI. In this study, exposure to repeated stress alone showed a transient increase in anxiety but no significant memory impairment or cellular and molecular changes. In contrast, repeated stress and blast resulted in lasting behavioral, molecular, and cellular abnormalities characterized by memory impairment, neuronal and glial cell loss, inflammation, and gliosis.

Listed below are examples of the most studied candidate protein biomarkers for TBI. These represent potential biomarkers of TBI that have shown high sensitivity and specificity in independent studies. UCHL1, SBDPs, and neuron-specific enolase (NSE) are presented as examples of neuronal and axonal protein biomarkers. For glial-specific markers, GFAP and S100beta are discussed below.

### Ubiquitin carboxy-terminal hydrolase L1 protein

Ubiquitin carboxy-terminal hydrolase L1 protein is a cysteine protease that is predominantly expressed in neurons, although it is also expressed in small amounts in neuroendocrine cells. This enzyme is relatively small, around 25 kDa and comprises *∼*2% of the total soluble protein in the brain. The other name for this protein is neuronal-specific protein gene product 9.5. Known function of UCHL1 is that it hydrolyzes the C-terminal bond of ubiquitin or unfolded polypeptides (Setsuie and Wada, 2007).

Several publications have indicated that UCHL1 can be a biomarker for TBI. Recently, the biokinetic parameters of UCHL1 were measured from a cohort of severely injured TBI patients (Brophy et al., 2011). A more recent study (Mondello et al., 2012b) demonstrated that UCHL1 can be used as a biomarker for severely injured TBI patients. Compared to control, the serum UCHL1 levels of TBI patients were significantly elevated measured after the acute phase and then over a week.

### Spectrin breakdown products

AlphaII-spectrin is primarily found in neurons and is concentrated in axons and presynaptic terminals (Riederer et al., 1986). Upon activation in TBI, calpain cleaves the protein to breakdown products (SBDPs) of molecular weights 150 kDa (SBDP150) and 145 kDa (SBDP145) and casapse-3 cleaves it to a 120-kDa product (SBDP120). Calpain and caspase-3 are major executioners of necrotic and apoptotic cell death, respectively, during ischemia or TBI (Ringger et al., 2004; Pineda et al., 2007; Mondello et al., 2010). SBDPs concurrently indicate calpain and caspase-3 proteolysis of alphaII-spectrin, providing crucial information on the underlying cell death mechanisms. In CSF, distinct temporal release patterns of SBDP145 and SBDP120 were observed to reflect different temporal characteristics of protease activation (Mondello et al., 2010). Elevated levels of SBDPs in CSF from adults with severe TBI were reported and their significant relationships with severity of injury and outcome (Pineda et al., 2007). Increased CSF SBDP levels were found to be significantly associated with mortality in patients with severe TBI. The temporal profile of SBDPs in non-survivors was also found to be different those of survivors (Mondello et al., 2010). Taken together, these findings suggest that SBDPs may provide crucial information not only on severity of brain injury, but also on underlying pathophysiological mechanisms associated with necrotic and apoptotic cell death.

### Neuron-specific enolase

Neuron-specific enolase is a glycolytic pathway enzyme and highly expressed in neuronal cytoplasm. NSE has been shown to have the sensitivity and specificity to detect neuronal cell death (Selakovic et al., 2005). In addition, studies have been conducted examining CSF and serum NSE levels from adults with severe TBI, and their relationship with severity of injury and clinical outcome. Increased CSF and serum levels of NSE have been reported after TBI. NSE concentrations were also associated with severity of injury, CT scan findings, and outcome (Ross et al., 1996; Herrmann et al., 2000; Selakovic et al., 2005).

### Glial fibrillary acidic protein

Of the numerous candidate biomarkers for TBI, this protein holds the most promise. One of the strengths of GFAP as an ideal biomarker for TBI is that this protein is not found outside the central nervous system (Galea et al., 1995). First reported in 1971 (Eng et al., 1971), GFAP is found only in astroglial cytoskeleton. GFAP is an intermediate filament protein that forms networks that support the astroglial cells. Damage to the astroglial cells (astrogliosis) shows subsequent upregulation of GFAP. During injury, astroglial cells react by producing more GFAP. Evidence shows that serum GFAP is elevated with several types of brain damage, including TBI (Pelinka et al., 2004a,b; Nylén et al., 2006).

What makes GFAP specific to brain trauma is that even if the body is subjected to multiple forms of trauma, GFAP doesn’t spike up without brain injury (Pelinka et al., 2004b; Vos et al., 2004). Thus, GFAP as a biomarker is a specific indicator of injury to the glia. There’s also a high likelihood that GFAP can predict death or unfavorable outcomes (Vos et al., 2010; Zurek and Fedora, 2012). According to the proceedings of the military mild TBI diagnostic workshop (2010), validation studies in humans are already on-going (Marion et al., 2011).

### S100ß

S100ß is mainly found in astroglia and Schwann cells (Donato, 1986; Donato et al., 1986a,b), and is one of the most well-known biomarkers of brain damage. The concentration of S100ß is known to increase in the CSF and serum after injury making this protein a potential biomarker for TBI (Townend et al., 2006). This protein is not influenced by hemolysis and has a biological half-life of 2 h. S100ß belongs to a family of low molecular weight (9–13 kDa) calcium-binding S100 proteins and is involved in signal transduction (Heizmann et al., 2002). Several studies have examined the value of this marker, demonstrating correlation with injury and outcome (Pelinka et al., 2003a; Berger et al., 2005; Kleindienst et al., 2007; Egea-Guerrero et al., 2012). However, several limitations have been found. First, S100ß is not specific to the brain, showing up in non-nervous cells such as adipocytes, epidermal, chondrocytes, melanoma cells, and Langerhans cells (Zimmer et al., 1995). The presence of this protein outside the central nervous system is compounded by the problem that general trauma without brain injury can increase the said protein (Rothoerl and Woertgen, 2001). Second, S100ß spikes up after hemorrhagic shock, correlating the concentration to shock severity (Pelinka et al., 2003a,b,c). Because of this, it seems that S100ß cannot be used as a single biomarker for TBI. A recent study has looked at the ratio of S100ß against GFAP (Pelinka et al., 2004a), instead of looking at S100ß alone. In the study, the ratio of GFAP against S100ß was used to determine brain damage and prognosis. In another study, S100ß seemed to be a useful indicator of patients with intracranial lesion (Egea-Guerrero et al., 2012).

Another limitation in using S100ß as a biomarker for TBI is the relatively short serum half-life (Jackson et al., 2000). The obvious countermeasure to this problem is to measure the proteins right after injury; however, most mild TBI victims are not evaluated as soon as the injury occurs.

## Conclusion

Proteomics, with the advancement in MS along with the bioinformatics software, has opened opportunities to interrogate protein dynamics and provide insights into the biochemistry of TBI. Over the past years, proteomics has led to the discovery of many candidate biomarkers and is becoming the method-of-choice for preliminary candidate marker selection. However, identification of candidate biomarkers using this approach is proving to be only the initial step in the development of a useful biomarker. Systems biology coupled to data mining strategies has been applied to harness these large data sets into organized and interlinked databases that can be queried to identify non-redundant brain injury pathways. The pathways can be exploited to determine the utilities of these proteins as diagnostic biomarkers and/or therapeutic targets.

This review provides an overview of the integration of proteomics, bioinformatics, and systems biology in TBI biomarker discovery. At present, proteomic biomarker discovery experiments have generated a long list of TBI biomarker candidates. Clearly, the next step is translating a robust biomarker or panel of biomarkers to clinical use. Currently, sensitive and specific immunoassays are being developed to validate a number of TBI biomarkers in clinical samples. However, the high cost of assay development and availability of antibodies result in a bottleneck in the clinical validation pipeline of the long list of discovered potential biomarkers. Targeted proteomics is a growing trend among the proteomic community. Mass spectrometry-based measurements such as multiple reaction monitoring (MRM) is a promising technique that could revolutionize biomarker validation. The current technologies are still evolving to address fundamental problems in identifying low abundant protein biomarkers such as in the case of mild TBI. The trend of lower costs, highly sensitive instruments (Orbitrap), and better electronic hardware will most likely increase targeted proteomics experiments in the future.

## Conflict of Interest Statement

The authors declare that the research was conducted in the absence of any commercial or financial relationships that could be construed as a potential conflict of interest.

## References

[B1] Agoston D. V. Gyorgy A. Eidelman O. Pollard H. B. (2009). Proteomic biomarkers for blast neurotrauma: targeting cerebral edema, inflammation, and neuronal death cascades. J. Neurotrauma 26, 901–911 10.1089/neu.2008.0724 19397421

[B2] Alexander M. P. (1995). Mild traumatic brain injury: pathophysiology, natural history, and clinical management. Neurology 45, 1253–1260 10.1212/WNL.45.7.1253 7617178

[B3] Alzate O. (ed.). (2010). “Neuroproteomics,” in Neuroproteomics, Chap. 1. Boca Raton: CRC Press

[B4] Appella E. Padlan E. A. Hunt D. F. (1995). Analysis of the structure of naturally processed peptides bound by class I and class II major histocompatibility complex molecules. EXS 73, 105–119 7579970 10.1007/978-3-0348-9061-8_6

[B5] Arciniegas D. B. Silver J. M. (2001). Regarding the search for a unified definition of mild traumatic brain injury. Brain Inj. 15, 649–652 10.1080/02699050010019800 11429094

[B6] Bairoch A. Apweiler R. (1997). The SWISS-PROT protein sequence database: its relevance to human molecular medical research. J. Mol. Med. 75, 312–316 9181472

[B7] Bairoch A. Boeckmann B. (1991). The SWISS-PROT protein sequence data bank. Nucleic Acids Res. 19, 2247–2249 10.1093/nar/19.suppl.2247 2041811 PMC331359

[B8] Bayes A. Grant S. G. (2009). Neuroproteomics: understanding the molecular organization and complexity of the brain. Nat. Rev. Neurosci. 10, 635–646 10.1038/nrn2701 19693028

[B9] Bencsath F. A. Field F. H. (1988). Ion retardation and collision-induced dissociation in the thermospray ion source. Anal. Chem. 60, 1323–1329 10.1021/ac00164a016 3213948

[B10] Benzinger T. L. Brody D. Cardin S. Curley K. C. Mintun M. A. Mun S. K. (2009). Blast-related brain injury: imaging for clinical and research applications: report of the 2008 St. Louis workshop. J. Neurotrauma 26, 2127–2144 10.1089/neu.2009-0885 19508154 PMC2824226

[B11] Berger R. P. Adelson P. D. Pierce M. C. Dulani T. Cassidy L. D. Kochanek P. M. (2005). Serum neuron-specific enolase, S100B, and myelin basic protein concentrations after inflicted and noninflicted traumatic brain injury in children. J. Neurosurg. 103, 61–68 16122007 10.3171/ped.2005.103.1.0061

[B12] Binder L. M. (1997). A review of mild head trauma. Part II: clinical implications. J. Clin. Exp. Neuropsychol. 19, 432–457 10.1080/01688639708403871 9268817

[B13] Bissonnette L. Bergeron M. G. (2006). Next revolution in the molecular theranostics of infectious diseases: microfabricated systems for personalized medicine. Expert Rev. Mol. Diagn. 6, 433–450 10.1586/14737159.6.3.433 16706745

[B14] Blackstock W. P. Weir M. P. (1999). Proteomics: quantitative and physical mapping of cellular proteins. Trends Biotechnol. 17, 121–127 10.1016/S0167-7799(98)01245-1 10189717

[B15] Boutté A. M. Yao C. Kobeissy F. May L. uX. C. Zhang Z. Wang K. K. (2012). Proteomic analysis and brain-specific systems biology in a rodent model of penetrating ballistic-like brain injury. Electrophoresis 33, 3693–3704. 10.1002/elps.201200196 23161467

[B16] Brophy G. M. Mondello S. Papa L. Robicsek S. A. Gabrielli A. Tepas J. III (2011). Biokinetic analysis of ubiquitin C-terminal hydrolase-L1 (UCH-L1) in severe traumatic brain injury patient biofluids. J. Neurotrauma 28, 861–870 10.1089/neu.2010.1564 21309726 PMC3113451

[B17] Büki A. Siman R. Trojanowski J. Q. Povlishock J. T. (1999). The role of calpain-mediated spectrin proteolysis in traumatically induced axonal injury. J. Neuropathol. Exp. Neurol. 58, 365–375 10.1097/00005072-199904000-00007 10218632

[B18] Carey M. E. (1996). Analysis of wounds incurred by U.S. Army Seventh Corps personnel treated in Corps hospitals during Operation Desert Storm, February 20 to March 10, 1991. J. Trauma 40, S165–S169 10.1097/00005373-199603001-00036 8606402

[B19] Chen X. Yu L. Steill J. D. Oomens J. Polfer N. C. (2009). Effect of peptide fragment size on the propensity of cyclization in collision-induced dissociation: oligoglycine b(2)-b(8). J. Am. Chem. Soc. 131, 18272–18282 10.1021/ja9030837 19947633

[B20] Cheng J. Gu J. Ma Y. Yang T. Kuang Y. Li B. (2010). Development of a rat model for studying blast-induced traumatic brain injury. J. Neurol. Sci. 294, 23–28 10.1016/j.jns.2010.04.010 20478573

[B21] Choudhary J. Grant S. G. (2004). Proteomics in postgenomic neuroscience: the end of the beginning. Nat. Neurosci. 7, 6 10.1038/nn1240 15114355

[B22] Chuang H. Y. Hofree M. Ideker T. (2010). A decade of systems biology. Annu. Rev. Cell Dev. Biol. 26, 721–744 10.1146/annurev-cellbio-100109-104122 20604711 PMC3371392

[B23] Colinge J. Bennett K. L. (2007). Introduction to computational proteomics. PLoS Comput. Biol. 3:e114 10.1371/journal.pcbi.0030114 17676979 PMC1933459

[B24] Crawford F. Crynen G. Reed J. Mouzon B. Bishop A. Katz B. (2012). Identification of plasma biomarkers of TBI outcome using proteomic approaches in an APOE mouse model. J. Neurotrauma 29, 246–260 10.1089/neu.2011.1789 21895520

[B25] Dixon C. E. Clifton G. L. Lighthall J. W. Yaghmai A. A. Hayes R. L. (1991). A controlled cortical impact model of traumatic brain injury in the rat. J. Neurosci. Methods 39, 253–262 10.1016/0165-0270(91)90104-8 1787745

[B26] Donato R. (1986). S-100 proteins. Cell Calcium 7, 123–145 10.1016/0143-4160(86)90017-5 3521884

[B27] Donato R. Battaglia F. Cocchia D. (1986a). Effects of S-100 proteins on assembly of brain microtubule proteins: correlation between kinetic and ultrastructural data. J. Neurochem. 47, 350–354 10.1111/j.1471-4159.1986.tb04508.x 3734782

[B28] Donato R. Prestagiovanni B. Zelano G. (1986b). Identity between cytoplasmic and membrane-bound S-100 proteins purified from bovine and rat brain. J. Neurochem. 46, 1333–1337 10.1111/j.1471-4159.1986.tb01743.x 3514791

[B29] Egea-Guerrero J. J. Revuelto-Rey J. Murillo-Cabezas F. Muñoz-Sánchez M. A. Vilches-Arenas A. Sánchez-Linares P. (2012). Accuracy of the S100beta protein as a marker of brain damage in traumatic brain injury. Brain Inj. 26, 76–82 10.3109/02699052.2011.635360 22149446

[B30] Elias J. E. Gygi S. P. (2010). Target-decoy search strategy for mass spectrometry-based proteomics. Methods Mol. Biol. 604, 55–71 10.1007/978-1-60761-444-9_5 20013364 PMC2922680

[B31] Eng L. F. Vanderhaeghen J. J. Bignami A. Gerstl B. (1971). An acidic protein isolated from fibrous astrocytes. Brain Res. 28, 351–354 10.1016/0006-8993(71)90668-8 5113526

[B32] Farkas O. Polgár B. Szekeres-Barthó J. Dóczi T. Povlishock J. T. Büki A. (2005). Spectrin breakdown products in the cerebrospinal fluid in severe head injury – preliminary observations. Acta Neurochir. (Wien) 147, 855–861 10.1007/s00701-005-0559-6 15924207

[B33] Fenn J. B. Mann M. Meng C. K. Wong S. F. Whitehouse C. M. (1989). Electrospray ionization for mass spectrometry of large biomolecules. Science 246, 64–71 10.1126/science.2675315 2675315

[B34] Frank A. Pevzner P. (2005). PepNovo: de novo peptide sequencing via probabilistic network modeling. Anal. Chem. 77, 964–973 10.1021/ac048788h 15858974

[B35] Fraser D. Powell R. E. (1950). The kinetics of trypsin digestion. J. Biol. Chem. 187, 803–820 14803465

[B36] Galea E. Dupouey P. Feinstein D. L. (1995). Glial fibrillary acidic protein mRNA isotypes: expression in vitro and in vivo. J. Neurosci. Res. 41, 452–461 10.1002/jnr.490410404 7473876

[B37] Gerberding J. (2003). Report to Congress on Mild Traumatic Brain Injury in the Ubited States: Steps to Prevent a Serious Public Health Problem. Atlanta: National Center for Injury Prevention and Control

[B38] Guingab-Cagmat J. D. Newsom K. Vakulenko A. Cagmat E. B. Kobeissy F. H. Zoltewicz S. (2012). An in vitro mass spectrometry-based proteomic analysis and absolute quantification of neuronal-glial injury biomarkers in cell culture system. Electrophoresis 33, 3786–3797 10.1002/elps.201200326 23161537

[B39] Hagen J. B. (2000). The origins of bioinformatics. Nat. Rev. Genet. 1, 231–236 10.1038/35042090 11252753

[B40] Hamm R. J. Lyeth B. G. Jenkins L. W. O’Dell D. M. Pike B. R. (1993). Selective cognitive impairment following traumatic brain injury in rats. Behav. Brain Res. 59, 169–173 10.1016/0166-4328(93)90164-L 8155285

[B41] Hanrieder J. Wetterhall M. Enblad P. Hillered L. Bergquist J. (2009). Temporally resolved differential proteomic analysis of human ventricular CSF for monitoring traumatic brain injury biomarker candidates. J. Neurosci. Methods 177, 469–478 10.1016/j.jneumeth.2008.10.038 19263575

[B42] Haqqani A. S. Hutchison J. S. Ward R. Stanimirovic D. B. (2007a). Biomarkers and diagnosis; protein biomarkers in serum of pediatric patients with severe traumatic brain injury identified by ICAT-LC-MS/MS. J. Neurotrauma 24, 54–74 10.1089/neu.2006.0079 17263670

[B43] Haqqani A. S. Kelly J. Baumann E. Haseloff R. F. Blasig I. E. Stanimirovic D. B. (2007b). Protein markers of ischemic insult in brain endothelial cells identified using 2D gel electrophoresis and ICAT-based quantitative proteomics. J. Proteome Res. 6, 226–239 10.1021/pr0603811 17203967

[B44] Heizmann C. W. Fritz G. Schafer B. W. (2002). S100 proteins: structure, functions and pathology. Front. Biosci. 7:d1356–d1368 10.2741/heizmann 11991838

[B45] Hergenroeder G. Redell J. B. Moore A. N. Dubinsky W. P. Funk R. T. Crommett J. (2008). Identification of serum biomarkers in brain-injured adults: potential for predicting elevated intracranial pressure. J. Neurotrauma 25, 79–93 10.1089/neu.2007.0386 18260791

[B46] Herrmann M. Jost S. Kutz S. Ebert A. D. Kratz T. Wunderlich M. T. (2000). Temporal profile of release of neurobiochemical markers of brain damage after traumatic brain injury is associated with intracranial pathology as demonstrated in cranial computerized tomography. J. Neurotrauma 17, 113–122 10.1089/neu.2000.17.113 10709869

[B47] Hinman L. M. Huang S. M. Hackett J. Koch W. H. Love P. Y. Pennello G. (2006). The drug diagnostic co-development concept paper: commentary from the 3rd FDA-DIA-PWG-PhRMA-BIO Pharmacogenomics Workshop. Pharmacogenomics J. 6, 375–380 10.1038/sj.tpj.6500392 16652120

[B48] Hooper J. W. (2006). The genetic map to theranostics. MLO Med. Lab. Obs. 38, 22–23 16838759

[B49] Ideker T. Galitski T. Hood L. (2001). A new approach to decoding life: systems biology. Annu. Rev. Genomics Hum. Genet. 2, 343–372 10.1146/annurev.genom.2.1.343 11701654

[B50] Inglese M. Makani S. Johnson G. Cohen B. A. Silver J. A. Gonen O. (2005). Diffuse axonal injury in mild traumatic brain injury: a diffusion tensor imaging study. J. Neurosurg. 103, 298–303 10.3171/jns.2005.103.2.0298 16175860

[B51] Jackson R. G. Samra G. S. Radcliffe J. Clark G. H. Price C. P. (2000). The early fall in levels of S-100 beta in traumatic brain injury. Clin. Chem. Lab. Med. 38, 1165–1167 10.1515/CCLM.2000.179 11156351

[B52] Jager T. E. Weiss H. B. Coben J. H. Pepe P. E. (2000). Traumatic brain injuries evaluated in U.S. emergency departments, 1992–1994. Acad. Emerg. Med. 7, 134–140 10.1111/j.1553-2712.2000.tb00515.x 10691071

[B53] Jenkins L. W. Peters G. W. Dixon C. E. Zhang X. Clark R. S. Skinner J. C. (2012). Conventional and functional proteomics using large format two-dimensional gel electrophoresis 24 hours after controlled cortical impact in postnatal day 17 rats. J. Neurotrauma 19, 715–740 10.1089/08977150260139101 12165133

[B54] Johnson E. A. Svetlov S. I. Pike B. R. Tolentino P. J. Shaw G. Wang K. K. (2004). Cell-specific upregulation of survivin after experimental traumatic brain injury in rats. J. Neurotrauma 21, 1183–1195 10.1089/neu.2004.21.1183 15453988

[B55] Karas M. Hillenkamp F. (1988). Laser desorption ionization of proteins with molecular masses exceeding 10,000 daltons. Anal. Chem. 60, 2299–2301 10.1021/ac00171a028 3239801

[B56] Kibby M. Y. Long C. J. (1996). Minor head injury: attempts at clarifying the confusion. Brain Inj. 10, 159–186 10.1080/026990596124494 8777389

[B57] Kitano H. (2002a). Systems biology: a brief overview. Science 295, 1662–1664 10.1126/science.1069492 11872829

[B58] Kitano H. (2002b). Computational systems biology. Nature 420, 206–210 10.1038/nature01254 12432404

[B59] Kleindienst A. Hesse F. Bullock M. R. Buchfelder M. (2007). “The neurotrophic protein S100B: value as a marker of brain damage and possible therapeutic implications,” in Progress in Brain Research, eds John T. W. Andrew I. R. M. (Amsterdam: Elsevier), 161, 317–325 10.1016/S0079-6123(06)61022-4 17618987

[B60] Klose J. Feller M. (1981). Genetic variability of proteins from plasma membranes and cytosols of mouse organs. Biochem. Genet. 19, 859–870 10.1007/BF00504251 7332527

[B61] Kobeissy F. H. Ottens A. K. Zhang Z. Liu M. C. Denslow N. D. Dave J. R. (2006). Novel differential neuroproteomics analysis of traumatic brain injury in rats. Mol. Cell Proteomics 5, 1887–1898 10.1074/mcp.M600157-MCP200 16801361

[B62] Kobeissy F. H. Sadasivan S. Oli M. W. Robinson G. Larner S. F. Zhang Z. (2008). Neuroproteomics and systems biology-based discovery of protein biomarkers for traumatic brain injury and clinical validation. Proteomics. Clin. Appl. 2, 1467–1483 10.1002/prca.200800011 21136795

[B63] Kumar C. Mann M. (2009). Bioinformatics analysis of mass spectrometry-based proteomics data sets. FEBS Lett. 583, 1703–1712 10.1016/j.febslet.2009.03.035 19306877

[B64] Kushner D. (1998). Mild traumatic brain injury: toward understanding manifestations and treatment. Arch. Intern. Med. 158, 1617–1624 10.1001/archinte.158.15.1617 9701095

[B65] Kwon S. K. Kovesdi E. Gyorgy A. B. Wingo D. Kamnaksh A. Walker J. (2011). Stress and traumatic brain injury: a behavioral, proteomics, and histological study. Front. Neurol. 2:12 10.3389/fneur.2011.00012 21441982 PMC3057553

[B66] Lesko L. J. Atkinson A. J. Jr. (2001). Use of biomarkers and surrogate endpoints in drug development and regulatory decision making: criteria, validation, strategies. Annu. Rev. Pharmacol. Toxicol. 41, 347–366 10.1146/annurev.pharmtox.41.1.347 11264461

[B67] Lew H. L. (2005). Rehabilitation needs of an increasing population of patients: traumatic brain injury, polytrauma, and blast-related injuries. J. Rehabil. Res. Dev. 42, xiii–xvi 10.1682/JRRD.2005.07.0124 16320135

[B68] Lighthall J. W. (1988). Controlled cortical impact: a new experimental brain injury model. J. Neurotrauma 5, 1–15 10.1089/neu.1988.5.1 3193461

[B69] Liu Z. Schey K. L. (2008). Fragmentation of multiply-charged intact protein ions using MALDI TOF-TOF mass spectrometry. J. Am. Soc. Mass Spectrom. 19, 231–238 10.1016/j.jasms.2007.06.006 17693096 PMC2288703

[B70] Lyeth B. G. Jenkins L. W. Hamm R. J. Dixon C. E. Phillips L. L. Clifton G. L. (1990). Prolonged memory impairment in the absence of hippocampal cell death following traumatic brain injury in the rat. Brain Res. 526, 249–258 10.1016/0006-8993(90)91229-A 2257484

[B71] Ma B. Zhang K. Hendrie C. Liang C. Li M. Doherty-Kirby A. (2003). PEAKS: powerful software for peptide de novo sequencing by tandem mass spectrometry. Rapid Commun. Mass Spectrom. 17, 2337–2342 10.1002/rcm.1196 14558135

[B72] Maggio E. T. Ramnarayan K. (2001). Recent developments in computational proteomics. Drug Discov. Today 6, 996–1004 10.1016/S1359-6446(01)02003-7 11576866

[B73] Management of Concussion/mTBI Working Group (2009). VA/DoD clinical practice guideline for management of concussion/mild traumatic brain injury. J. Rehabil. Res. Dev. 46, C1–68 10.1682/JRRD.2008.03.0038 20108447

[B74] Mann M. Wilm M. (1994). Error-tolerant identification of peptides in sequence databases by peptide sequence tags. Anal. Chem. 66, 4390–4399 10.1021/ac00096a002 7847635

[B75] Margulies S. (2000). The postconcussion syndrome after mild head trauma part II: is migraine underdiagnosed? J. Clin. Neurosci. 7, 495–499 10.1054/jocn.1999.0773 11029228

[B76] Marion D. W. Curley K. C. Schwab K. Hicks R. R. mTBI Diagnostics Workgroup (2011). Proceedings of the military mTBI Diagnostics Workshop, St. Pete Beach, August 2010. J. Neurotrauma 28, 517–526 10.1089/neu.2010.1638 21265587

[B77] Marshall A. G. Hendrickson C. L. Jackson G. S. (1998). Fourier transform ion cyclotron resonance mass spectrometry: a primer. Mass Spectrom. Rev. 17, 1–35 10.1002/(SICI)1098-2787(1998)17:1<1::AID-MAS1>3.0.CO;2-K 9768511

[B78] Mathers C. D. Loncar D. (2006). Projections of global mortality and burden of disease from 2002 to 2030. PLoS Med. 3:e442 10.1371/journal.pmed.0030442 17132052 PMC1664601

[B79] Matthiesen R. (2007). Methods, algorithms and tools in computational proteomics: a practical point of view. Proteomics 7, 2815–2832 10.1002/pmic.200700116 17703506

[B80] McDonald W. H. Yates J. R. III (2002). Shotgun proteomics and biomarker discovery. Dis. Markers 18, 99–105 12364816 10.1155/2002/505397 PMC3851423

[B81] McDonald W. H. Yates J. R. III (2003). Shotgun proteomics: integrating technologies to answer biological questions. Curr. Opin. Mol. Ther. 5, 302–309 12870441

[B82] Merril C. R. Goldman D. Van Keuren M. L. (1983). Silver staining methods for polyacrylamide gel electrophoresis. Meth. Enzymol. 96, 230–239 10.1016/S0076-6879(83)96021-4 6197605

[B83] Mondello S. Jeromin A. Buki A. Bullock R. Czeiter E. Kovacs N. (2012a). Glial neuronal ratio: a novel index for differentiating injury type in patients with severe traumatic brain injury. J. Neurotrauma 29, 1096–1104 10.1089/neu.2011.2092 22165978 PMC3325554

[B84] Mondello S. Linnet A. Buki A. Robicsek S. Gabrielli A. Tepas J. (2012b). Clinical utility of serum levels of ubiquitin C-terminal hydrolase as a biomarker for severe traumatic brain injury. Neurosurgery 70, 666–675 10.1227/NEU.0b013e318236a809 21937927 PMC3288385

[B85] Mondello S. Robicsek S. A. Gabrielli A. Brophy G. M. Papa L. Tepas J. (2010). AlphaII-spectrin breakdown products (SBDPs): diagnosis and outcome in severe traumatic brain injury patients. J. Neurotrauma 27, 1203–1213 10.1089/neu.2010.1278 20408766 PMC2942904

[B86] Niogi S. N. Mukherjee P. (2010). Diffusion tensor imaging of mild traumatic brain injury. J. Head Trauma Rehabil. 25, 241–255 10.1097/HTR.0b013e3181e52c2a 20611043

[B87] Nylén K. Ost M. Csajbok L. Z. Nilsson I. Blennow K. Nellgård B. (2006). Increased serum-GFAP in patients with severe traumatic brain injury is related to outcome. J. Neurol. Sci. 240, 85–91 10.1016/j.jns.2005.09.007 16266720

[B88] O’Farrell P. H. (1975). High resolution two-dimensional electrophoresis of proteins. J. Biol. Chem. 250, 4007–4021 236308 PMC2874754

[B89] Okie S. (2005). Traumatic brain injury in the war zone. N. Engl. J. Med. 352, 2043–2047 10.1056/NEJMp058102 15901856

[B90] Opii W. O. Nukala V. N. Sultana R. Pandya J. D. Day K. M. Merchant M. L. (2007). Proteomic identification of oxidized mitochondrial proteins following experimental traumatic brain injury. J. Neurotrauma 24, 772–789 10.1089/neu.2006.0229 17518533

[B91] Ottens A. K. Bustamante L. Golden E. C. Yao C. Hayes R. L. Wang K. K. (2010). Neuroproteomics: a biochemical means to discriminate the extent and modality of brain injury. J. Neurotrauma 27, 1837–1852 10.1089/neu.2010.1374 20698760 PMC2953930

[B92] Ottens A. K. Kobeissy F. H. Fuller B. F. Liu M. C. Oli M. W. Hayes R. L. (2007). Novel neuroproteomic approaches to studying traumatic brain injury. Prog. Brain Res. 161, 401–418 10.1016/S0079-6123(06)61029-7 17618994

[B93] Ottens A. K. Kobeissy F. H. Golden E. C. Zhang Z. Haskins W. E. Chen S. S. (2006). Neuroproteomics in neurotrauma. Mass Spectrom. Rev. 25, 380–408 10.1002/mas.20073 16498609

[B94] Paizs B. Mann M. (2012). 23rd Sanibel Conference on mass spectrometry: from fragmentation mechanisms to sequencing: Tandem Mass Spectrometry based peptide and protein identification. J. Am. Soc. Mass Spectrom. 23, 575–576 10.1007/s13361-012-0358-2 22359095

[B95] Peitsch M. C. Wilkins M. R. Tonella L. Sanchez J. C. Appel R. D. Hochstrasser D. F. (1997). Large-scale protein modelling and integration with the SWISS-PROT and SWISS-2DPAGE databases: the example of *Escherichia coli*. Electrophoresis 18, 498–501 10.1002/elps.1150180326 9150930

[B96] Pelinka L. E. Kroepfl A. Leixnering M. Buchinger W. Raabe A. Redl H. (2004a). GFAP versus S100B in serum after traumatic brain injury: relationship to brain damage and outcome. J. Neurotrauma 21, 1553–1561 10.1089/neu.2004.21.1553 15684648

[B97] Pelinka L. E. Kroepfl A. Schmidhammer R. Krenn M. Buchinger W. Redl H. (2004b). Glial fibrillary acidic protein in serum after traumatic brain injury and multiple trauma. J. Trauma 57, 1006–1012 10.1097/01.TA.0000108998.48026.C3 15580024

[B98] Pelinka L. E. Toegel E. Mauritz W. Redl H. (2003a). Serum S 100 B: a marker of brain damage in traumatic brain injury with and without multiple trauma. Shock 19, 195–200 10.1097/00024382-200303000-00001 12630517

[B99] Pelinka L. E. Szalay L. Jafarmadar M. Schmidhammer R. Redl H. Bahrami S. (2003b). Circulating S100B is increased after bilateral femur fracture without brain injury in the rat. Br. J. Anaesth. 91, 595–597 10.1093/bja/aeg225 14504167

[B100] Pelinka L. E. Bahrami S. Szalay L. Umar F. Redl H. (2003c). Hemorrhagic shock induces an S 100 B increase associated with shock severity. Shock 19, 422–426 10.1097/01.shk.0000055345.58165.52 12744484

[B101] Perkins D. N. Pappin D. J. Creasy D. M. Cottrell J. S. (1999). Probability-based protein identification by searching sequence databases using mass spectrometry data. Electrophoresis 20, 3551–3567 10.1002/(SICI)1522-2683(19991201)20:18<3551::AID-ELPS3551>3.0.CO;2-2 10612281

[B102] Pineda J. A. Lewis S. B. Valadka A. B. Papa L. Hannay H. J. Heaton S. C. (2007). Clinical significance of alphaII-spectrin breakdown products in cerebrospinal fluid after severe traumatic brain injury. J. Neurotrauma 24, 354–366 10.1089/neu.2006.003789 17375999

[B103] Polfer N. C. Oomens J. Suhai S. Paizs B. (2005). Spectroscopic and theoretical evidence for oxazolone ring formation in collision-induced dissociation of peptides. J. Am. Chem. Soc. 127, 17154–17155 10.1021/ja056553x 16332041

[B104] Riederer B. M. Zagon I. S. Goodman S. R. (1986). Brain spectrin(240/235) and brain spectrin(240/235E): two distinct spectrin subtypes with different locations within mammalian neural cells. J. Cell Biol. 102, 2088–2097 10.1083/jcb.102.6.2088 3519621 PMC2114251

[B105] Rifai N. Gillette M. A. Carr S. A. (2006). Protein biomarker discovery and validation: the long and uncertain path to clinical utility. Nat. Biotechnol. 24, 971–983 10.1038/nbt1235 16900146

[B106] Ringger N. C. O’Steen B. E. Brabham J. G. Silver X. Pineda J. Wang K. K. (2004). A novel marker for traumatic brain injury: CSF alphaII-spectrin breakdown product levels. J. Neurotrauma 21, 1443–1456 10.1089/neu.2004.21.1443 15672634

[B107] Risdall J. E. Menon D. K. (2011). Traumatic brain injury. Philos. Trans. R. Soc. Lond. B Biol. Sci. 366, 241–250 10.1098/rstb.2010.0230 21149359 PMC3013429

[B108] Risling M. Plantman S. Angeria M. Rostami E. Bellander B. M. Kirkegaard M. (2011). Mechanisms of blast induced brain injuries, experimental studies in rats. Neuroimage 54, S89–97 10.1016/j.neuroimage.2010.05.031 20493951

[B109] Roepstorff P. Fohlman J. (1984). Proposal for a common nomenclature for sequence ions in mass spectra of peptides. Biomed. Mass Spectrom. 11, 601 10.1002/bms.1200111109 6525415

[B110] Ross S. A. Cunningham R. T. Johnston C. F. Rowlands B. J. (1996). Neuron-specific enolase as an aid to outcome prediction in head injury. Br. J. Neurosurg. 10, 471–476 10.1080/02688699647104 8922706

[B111] Rothoerl R. D. Woertgen C. (2001). High serum S100B levels for trauma patients without head injuries. Neurosurgery 49, 1490–1491 10.1097/00006123-200112000-00053 11859837

[B112] Ruff R. M. Jurica P. (1999). In search of a unified definition for mild traumatic brain injury. Brain Inj. 13, 943–952 10.1080/026990599120963 10628500

[B113] Selakovic V. Raicevic R. Radenovic L. (2005). The increase of neuron-specific enolase in cerebrospinal fluid and plasma as a marker of neuronal damage in patients with acute brain infarction. J. Clin. Neurosci. 12, 542–547 10.1016/j.jocn.2004.07.019 15921910

[B114] Setsuie R. Wada K. (2007). The functions of UCH-L1 and its relation to neurodegenerative diseases. Neurochem. Int. 51, 105–111 10.1016/j.neuint.2007.05.007 17586089

[B115] Shadforth I. Crowther D. Bessant C. (2005). Protein and peptide identification algorithms using MS for use in high-throughput, automated pipelines. Proteomics 5, 4082–4095 10.1002/pmic.200402091 16196103

[B116] Shenton M. E. Hamoda H. M. Schneiderman J. S. Bouix S. Pasternak O. Rathi Y. (2012). A review of magnetic resonance imaging and diffusion tensor imaging findings in mild traumatic brain injury. Brain Imaging Behav. 6, 137–192 10.1007/s11682-012-9156-5 22438191 PMC3803157

[B117] Shevchenko A. Sunyaev S. Loboda A. Shevchenko A. Bork P. Ens W. (2001). Charting the proteomes of organisms with unsequenced genomes by MALDI-quadrupole time-of-flight mass spectrometry and BLAST homology searching. Anal. Chem. 73, 1917–1926 10.1021/ac0013709 11354471

[B118] Shi S. D. Hendrickson C. L. Marshall A. G. (1998). Counting individual sulfur atoms in a protein by ultrahigh-resolution Fourier transform ion cyclotron resonance mass spectrometry: experimental resolution of isotopic fine structure in proteins. Proc. Natl. Acad. Sci. U.S.A. 95, 11532–11537 10.1073/pnas.95.20.11532 9751700 PMC21675

[B119] Shoemaker L. D. Achrol A. S. Sethu P. Steinberg G. K. Chang S. D. (2012). Clinical neuroproteomics and biomarkers: from basic research to clinical decision making. Neurosurgery 70, 518–525 10.1227/NEU.0b013e3182333a26 21866062

[B120] Siman R. McIntosh T. K. Soltesz K. M. Chen Z. Neumar R. W. Roberts V. L. (2004). Proteins released from degenerating neurons are surrogate markers for acute brain damage. Neurobiol. Dis. 16, 311–320 10.1016/j.nbd.2004.03.016 15193288

[B121] Smith D. H. Uryu K. Saatman K. E. Trojanowski J. Q. McIntosh T. K. (2003). Protein accumulation in traumatic brain injury. Neuromolecular Med. 4, 59–72 10.1385/NMM:4:1-2:59 14528053

[B122] Stults J. T. Arnott D. (2005). Proteomics. Meth. Enzymol. 402, 245–289 10.1016/S0076-6879(05)02008-2 16401512

[B123] Svetlov S. I. Larner S. F. Kirk D. R. Atkinson J. Hayes R. L. Wang K. K. (2009). Biomarkers of blast-induced neurotrauma: profiling molecular and cellular mechanisms of blast brain injury. J. Neurotrauma 26, 913–921 10.1089/neu.2008.0609 19422293 PMC6469534

[B124] Svetlov S. I. Prima V. Kirk D. R. Gutierrez H. Curley K. C. Hayes R. L. (2010). Morphologic and biochemical characterization of brain injury in a model of controlled blast overpressure exposure. J. Trauma 69, 795–804 10.1097/TA.0b013e3181bbd885 20215974

[B125] Szczygielski J. Mautes A. Steudel W. I. Falkai P. Bayer T. A. Wirths O. (2005). Traumatic brain injury: cause or risk of Alzheimer’s disease? A review of experimental studies. J. Neural Transm. 112, 1547–1564 10.1007/s00702-005-0326-0 15959838

[B126] Townend W. Dibble C. Abid K. Vail A. Sherwood R. Lecky F. (2006). Rapid elimination of protein S-100B from serum after minor head trauma. J. Neurotrauma 23, 149–155 10.1089/neu.2006.23.149 16503799

[B127] Vos P. E. Jacobs B. Andriessen T. M. Lamers K. J. Borm G. F. Beems T. (2010). GFAP and S100B are biomarkers of traumatic brain injury: an observational cohort study. Neurology 75, 1786–1793 10.1212/WNL.0b013e3181fd62d2 21079180

[B128] Vos P. E. Lamers K. J. Hendriks J. C. van H. aaren M. Beems T. Zimmerman C. (2004). Glial and neuronal proteins in serum predict outcome after severe traumatic brain injury. Neurology 62, 1303–1310 10.1212/01.WNL.0000120550.00643.DC 15111666

[B129] Wang K. K. W. Ottens A. K. Liu M. C. Lewis S. B. Meegan C. Oli M. W. (2005). Proteomic identification of biomarkers of traumatic brain injury. Expert Rev. Proteomics, 2, 603–614 10.1586/14789450.2.4.603 16097892

[B130] Wang H. Qian W. J. Chin M. H. Petyuk V. A. Barry R. C. Liu T. (2006). Characterization of the mouse brain proteome using global proteomic analysis complemented with cysteinyl-peptide enrichment. J. Proteome Res. 5, 361–369 10.1021/pr0503681 16457602 PMC1850945

[B131] Warden D. (2006). Military TBI during the Iraq and Afghanistan wars. J. Head Trauma Rehabil. 21, 398–402 10.1097/00001199-200609000-00004 16983225

[B132] Wasinger V. C. Cordwell S. J. Cerpa-Poljak A. Yan J. X. Gooley A. A. Wilkins M. R. (1995). Progress with gene-product mapping of the Mollicutes: *Mycoplasma genitalium*. Electrophoresis 16, 1090–1094 10.1002/elps.11501601185 7498152

[B133] Webb-Robertson B. J. Cannon W. R. (2007). Current trends in computational inference from mass spectrometry-based proteomics. Brief. Bioinformatics 8, 304–317 10.1093/bib/bbm023 17584764

[B134] Williams A. J. Hartings J. A. Lu X. C. Rolli M. L. Dave J. R. Tortella F. C. (2005). Characterization of a new rat model of penetrating ballistic brain injury. J. Neurotrauma 22, 313–331 10.1089/neu.2005.22.313 15716636

[B135] Wolters D. A. Washburn M. P. Yates J. R. III. (2001). An automated multidimensional protein identification technology for shotgun proteomics. Anal. Chem. 73, 5683–5690 10.1021/ac010617e 11774908

[B136] Wu C. C. MacCoss M. J. (2002). Shotgun proteomics: tools for the analysis of complex biological systems. Curr. Opin. Mol. Ther. 4, 242–250 12139310

[B137] Xenarios I. Fernandez E. Salwinski L. Duan X. J. Thompson M. J. Marcotte E. M. (2001). DIP: the database of interacting proteins: 2001 update. Nucleic Acids Res. 29, 239–241 10.1093/nar/29.1.239 11125102 PMC29798

[B138] Xiong Y. Mahmood A. Chopp M. (2013). Animal models of traumatic brain injury. Nat. Rev. Neurosci. 14, 128–142 10.1038/nrn3407 23329160 PMC3951995

[B139] Xu C. Ma B. (2006). Software for computational peptide identification from MS-MS data. Drug Discov. Today 11, 595–600 10.1016/j.drudis.2006.05.011 16793527

[B140] Yates J. R. 3rd Eng J. K. McCormack A. L. Schieltz D. (1995). Method to correlate tandem mass spectra of modified peptides to amino acid sequences in the protein database. Anal. Chem. 67, 1426–1436 10.1021/ac00104a020 7741214

[B141] Zhang Z. Larner S. F. Kobeissy F. Hayes R. L. Wang K. K. (2010). Systems biology and theranostic approach to drug discovery and development to treat traumatic brain injury. Methods Mol. Biol. 662, 317–329 10.1007/978-1-60761-800-3_16 20824479

[B142] Zimmer D. B. Cornwall E. H. Landar A. Song W. (1995). The S100 protein family: history, function, and expression. Brain Res. Bull. 37, 417–429 10.1016/0361-9230(95)00040-2 7620916

[B143] Zurek J. Fedora M. (2012). The usefulness of S100B, NSE, GFAP, NF-H, secretagogin and Hsp70 as a predictive biomarker of outcome in children with traumatic brain injury. Acta Neurochir. (Wien) 154, 93–103 10.1007/s00701-011-1175-2 21976236

